# Genetic and Clinical Characteristics of Patients With Homozygous and Compound Heterozygous Familial Hypercholesterolemia From Three Different Populations: Case Series

**DOI:** 10.3389/fgene.2020.572176

**Published:** 2020-09-11

**Authors:** Tatiana Marusic, Ursa Sustar, Fouzia Sadiq, Vjosa Kotori, Matej Mlinaric, Jernej Kovac, Saeed Shafi, Iqbal Khan, Matija Cevc, Katarina Trebusak Podkrajsek, Tadej Battelino, Urh Groselj

**Affiliations:** ^1^University Children’s Hospital, University Medical Center, Ljubljana, Slovenia; ^2^Shifa Tameer-e-Millat University, Islamabad, Pakistan; ^3^Department of Endocrinology, Pediatric Clinic, University Clinical Center of Kosovo, Pristina, Kosovo; ^4^Department of Anatomy, Shifa Tameer-e-Millat University, Islamabad, Pakistan; ^5^Department of Vascular Surgery, Shifa International Hospital, Islamabad, Pakistan; ^6^Division of Medicine, Centre for Preventive Cardiology, University Medical Centre Ljubljana, Ljubljana, Slovenia; ^7^Faculty of Medicine, University of Ljubljana, Ljubljana, Slovenia

**Keywords:** familial hypercholesterolaemia, FH, homozygous, compound heterozygous, LDLR gene

## Abstract

Homozygous familial hypercholesterolemia (HoFH) and compound heterozygous familial hypercholesterolemia (cHeFH) are rare disorders generated by disease-causing variants in both alleles of the *LDLR* or other familial hypercholesterolemia (FH)-related genes. HoFH and cHeFH are characterized by severely elevated low-density lipoprotein-cholesterol (LDL-C), frequently leading to early cardiovascular disease. We investigated the genetic and clinical characteristics of HoFH and cHeFH patients from the Slovenian FH registry and/or those who were previously diagnosed or managed at our institution (Slovenian, Pakhtun and Albanian ethnicity), where genetic testing is not available. Our study includes seven patients. Their median age at the time of clinical diagnosis was 6.3 years (2.9–12.9 years); 2/7 were females. Two patients were diagnosed through the universal FH screening and five patients were diagnosed due to the presence of xanthomas. All the mutations are present in *LDLR* gene: 7 different genotypes for HoFH (p.Cys167Leu, p.Asp178Asn, p.Cys243Tyr, p.Gly549Asp, p.Cys27Trp, p.Ile585Thr and p.Val797Met) and p.Gly549Asp/p.Gln384Pro genotype for cHeFH patient. The median initial level of LDL-C was 17.0 mmol/L [655 mg/dL] (range 7.6–21.6 mmol/L). The HoFH/cHeFH patients are clinically and genetically very diverse. The clinical criteria (as Simon Broome criteria) might be applicable already in children to raise suspicion of FH but in some cases fail to distinguish heterozygous FH and HoFH/cHeFH patients. However, genetic testing is helpful in confirming the diagnosis, also for a prompt awareness, better compliance to treatment and family screening.

## Introduction

Familial Hypercholesterolemia (FH) is an autosomal dominant genetic disorder, characterized by elevated total cholesterol (TC) and LDL-cholesterol (LDL-C) levels, usually accompanied by clinical characteristics and an early onset of cardiovascular disease (CVD), with a variable severity depending on the causative mutation (Moorjani et al., 1993). The prevalence of homozygous FH is unknown because of underdiagnosis and of a wide spectrum of phenotypes overlapping with heterozygous FH (HeFH), but is estimated in 1:200,000–300,000 (Sjouke et al., 2015; EAS Familial Hypercholesterolaemia Studies Collaboration et al., 2018; Representatives of the Global Familial Hypercholesterolemia Community et al., 2020).

Homozygous FH can be classified as: a- Real homozygous (HoFH): when the same mutation affects both alleles of one of the major FH-related genes (*LDLR*, *APOB*, or *PCSK9*); b- Compound heterozygous (cHeFH): when different mutations located on different alleles affect one of the major FH-associated genes; c- Combined heterozygous: when different mutations affect two different FH-related genes (Masana et al., 2019).

Homozygous FH patients can develop xanthomas and progressive atherosclerosis already in early childhood. If untreated, they develop vascular lesions and CVD before the second decade of life and die before the end of the third decade (Watts et al., 2014; Vallejo-Vaz et al., 2015).

Slovenia has successfully implemented nationwide universal screening for FH in pre-school children, detecting also the HoFH/cHeFH patients (Klancar et al., 2015; Groselj et al., 2018). The implementation and optimization of the routine FH genetic testing enables us to offer it to other national and foreign centers.

We aimed to analyze characteristics of all patients with HoFH and cHeFH genetically diagnosed and/or managed at our center.

## Materials and Methods

In 2011, routine FH genetic diagnosis was introduced at the UMC – University Children’s Hospital Ljubljana, which serves as the national accredited genetic testing facility for dyslipidemias (Klancar et al., 2015; Groselj et al., 2018).

The universal hypercholesterolemia screening in children is an obligatory part of the blood check-up at the programed visit in 5-year old children at the primary care pediatricians; if TC is elevated (over 6 mmol/L or, in case of positive family history, over 5 mmol/L), the child is referred to the tertiary center for the FH genetic screening (Groselj et al., 2018).

We also provide FH genetic testing to other national and international institutions. Recently, we have been receiving samples from Pakistan and Kosovo (where the major ethnic group is Albanian). The first is a multiracial nation with a very heterogeneous population and without geographical clustering, generally found in other populations (Ajmal et al., 2010; Ahmed et al., 2013). A few common/known mutations are identified but the rest are mainly new mutations (Khan et al., 2014). On the other hand, a study in 2009 of FH in the Albanian population showed a single common mutation in almost half of the probands, but no other recent studies have been published (Diakou et al., 2010). Both populations require efficient genetic testing methods and a laboratory strategy.

The principles of the Declaration of Helsinki were followed and the Slovenian National Medical Ethics Committee (NMEC) approved the study (#25/12/10, #63/07/13 and 0120-273/2019/9). The data was also obtained from the National registry of FH and rare dyslipidemias (NMEC #0120-14/2017/5). Informed consent was obtained from adult patients and parents or legal guardians of minors.

The underlying data is partly available in the Mendeley repository at https://data.mendeley.com/datasets/thpt9htws6/1. The prevalence of HoFH/cHeFH in the Slovenian cohort was calculated as the number of live-born children since the implementation of the universal FH screening program in 1995, divided by the number of the HoFH/cHeFH patients.

### Genetic Analysis

By the beginning of 2020, around 1150 genetic analyses were performed on pediatric patients with hypercholesterolemia in our genetic laboratory. Genomic DNA was isolated from the patients whole blood samples using the FlexiGene isolation kit (Qiagen, Germany). Three different sequencing methods for FH gene detection were used over time: (1) targeted Sanger sequencing (*n* = 192) -LDLR gene and part of exon 26 of APOB gene-, (2) ADH MASTR v2 ready to use next-generation sequencing (NGS) based molecular assay (Multiplicom, Belgium) (*n* = 190)-for detection of the variants in coding regions of LDLR, PCSK9, APOE, part of exon 26 (c.10200 to c.11100) of I (Single Nucleotide Variants & Copy Number Variants), as well as 12 LDL-C raising SNPs for a comprehensive analysis-, and (3) xGen^®^ Lockdown^®^ NGS Probes (IDT, United States) (*n* = 652) -for an extended dyslipidemia panel (*APOB, LDLR, PCSK9, LDLRAP1*) and expanded dyslipidemia panel (*ABCA1, ABCG5, ABCG8, ALMS1, APOA1, APOA5, APOC2, APOC3, APOE, CREB3L3, GPIHBP1, LDLRAP1, LIPA, LMF1, LPL*)-. Samples were sequenced on MiSeq sequencer with MiSeq Reagent Kit (Illumina, United States) following the manufacturer’s protocol including recommendations for quality control parameters. In all samples sequenced with NGS, more than 100-fold horizontal coverage of the regions of interest (ROI) was achieved. A disease-specific database was used for determining the residual activity information of the known variants (Benito-Vicente et al., 2018). All variants found by the NGS were confirmed by targeted Sanger DNA sequencing.

## Case Series

**Patient 1** is a 13 years old boy of Pakhtun origin from Khyber Pakhtunkhwa Province of Pakistan. Two siblings of the patient had died prematurely at ages of 10 and 11 years; only after that the parents sought clinical help. He was clinically diagnosed with FH at the age of 11 because of xanthomas, corneal arcus and a TC level of 22.9 mmol/L [885 mg/dL]. The genetic testing showed three different mutations -p.Cys167Leu, p.Asp178Asn and p.Cys243Tyr- in both alleles (HoFH). All family members, except one sister, presented the same mutations in one allele (HeFH). To date, no functional data have been reported about the LDLR activity for these three variants. The patient presented a normal CT coronary angiography and coronary angiogram. He has been treated with atorvastatin 40 mg and ezetimibe 10 mg; other therapeutic options are not available in Pakistan.

**Patient 2** is a 14 years old male from Kosovo. Before the age of 3 years, his physician observed xanthomas on elbows and knees, suspecting FH. The lipid profile showed cholesterol levels more than 20 mmol/L (No more biochemical data is available from that country). The family history revealed hypercholesterolemia in both parents, brother and grandmother. He was initially treated in Kosovo with atorvastatin and underwent occasional LDL-apheresis, without any improvement. At 11 years of age, he was referred to Slovenia, confirming a homozygous mutation in the *LDLR*, with less than 2% of receptor activity. In the clinical center, the initial TC level was 15.8 mmol/L [610 mg/dL] and LDL-C level was 13.7 mmol/L [529 mg/dL]. At that moment he received atorvastatin 40 mg and ezetimibe 10 mg. The last carotid Ultrasound (US) at the age of 11 years revealed diffuse non-obstructive atherosclerosis in bilateral carotid arteries; the most significant plaque-type I-II in the right common carotid artery contributing to 30% stenosis. After that, he has continued with atorvastatin 80 mg, ezetimibe 10 mg and *PCSK9* inhibitors within a clinical trial, but was a non-responder to the later. Other therapeutic options are currently not available in Kosovo.

**Patient 3,** a 6 years old Slovenian girl (of Albanian origin), was recently diagnosed at the universal FH screening, with an initial TC level of 7.6 mmol/L [293 mg/dL]. She was referred to our tertiary care center, where the biochemical test was repeated and later the genetic test revealed an *LDLR* homozygous mutation with 15–30% of LDLR activity. She stays asymptomatic and no family history of CVD was found. Until now she had not been introduced to pharmacotherapy.

**Patient 4** is a 18 years old Slovenian male (of Albanian origin), diagnosed during the universal FH screening with TC level of 9.0 mmol/L [348 mg/dL]. His parents and brother have hypercholesterolemia. The genetic testing confirmed a homozygous *LDLR* mutation with 15–30% of LDLR residual activity. The last carotid US at the age of 16 years had shown a borderline intima-media thickness (cITM) of 0.56 mm. He started with monotherapy of atorvastatin and later required dual therapy with higher doses of atorvastatin and ezetimibe. At the age of 16 he was enrolled in the clinical trial with *PCSK9* inhibitors. His latest TC and LDL-C levels were 6 mmol/L [232 mg/dL] and 4.5 mmol/L [174 mg/dL], respectively.

**Patient 5** is a 31 years old Slovenian male with FH diagnosed in april 1993, at the age of 4, due to the presence of xanthomas. Both parents and sister have HeFH. His initial TC level was 24.8 mmol/L [959 mg/dL]. The genetic testing confirmed a homozygous mutation in *LDLR.* To date, no functional data were found about LDLR residual activity for this mutation. The last carotid US studies at the age of 30 years have shown a thickened intima-media (cITM 0.903 mm). He was treated with atorvastatin, LDL-apheresis and simvastatin to no effect. Finally, he required liver transplantation at the age of 16, with remarkable decrease of TC and LDL levels.

**Patient 6** is a 43 years old Slovene female clinically diagnosed with hypercholesterolemia at the age of 12 because of a strong family history and an elevated TC (more than 19 mmol/L). Her father died at 53 years old because of myocardial infarction and her mother also presented with coronary heart disease after the age of 60. She had a bad compliance to the combined therapy to atorvastatin 40 mg and ezetimibe 10 mg. She was receiving LDL-apheresis for several years (data about frequency of treatment unavailable), discontinued it later because of the adverse effects (strong headache, dizziness, weight gain, cough). When she was 38 years old she suffered an acute coronary syndrome, whereas coronary angiography revealed stenosis of the left main coronary artery and distal right coronary artery. She was diagnosed with homozygous mutation in *LDLR*, with an unknown residual activity. She transiently received PCSK9 inhibitor and lomitapide, but had discontinued the treatments due to side effects.

**Patient 7** is a 7 years old male from Kosovo. He was diagnosed with FH at the age of 6 after an abdominal pain event. At the examination, the physician discovered small xanthomas in the sacral region and the biochemical testing showed CT levels of 20.4 mmol/L [789 mg/dL]. His mother also presented hypercholesterolemia. The genetic analysis confirmed cHeFH; a variant in p.Gly549Asp, with less than 2% of LDLR residual activity, and another in p.Gln384Pro, with unknown residual activity. He has a normal carotid US. His current therapy consists of atorvastatin 80 mg and ezetimibe 10 mg and he is showing good response to the therapy. Other therapeutic options are not available in Kosovo.

**FIGURE 1:**
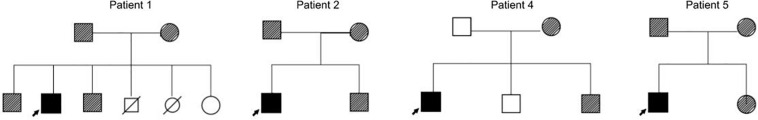
Pedigree charts from patients 1, 2, 4, and 5. 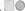: Heterozygous FH. 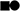: Homozygous FH.

**FIGURE 2:**
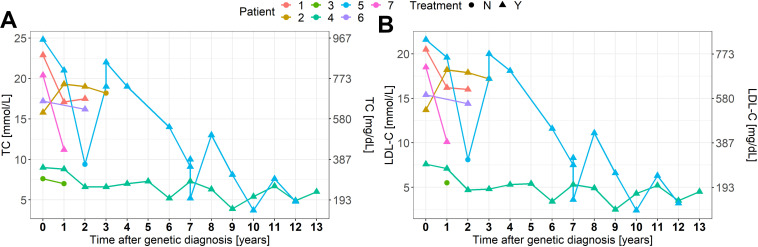
**(A,B)** TC and LDL-C levels in response to therapy. N, no; Y, yes.

Clinical and genetic features are summarized in Table 1 and Figure 1. Biochemical values and therapy are plotted in Figures 2A,B.

**TABLE 1:** Phenotypic and genotypic features of our cohort of HoFH and cHeFH patients.

Patient		1	2	3	4	5	6	7
**Age (years)**		13.0	14.0	6.9	18.5	31.6	43.0	7.9
**Gender**		M	M	F	M	M	F	M
**Ethnicity**		Pakhtun	Albanian	Albanian	Albanian	Slovene	Slovene	Albanian
Genetic desorder	Zygosity	**homozygous**	**homozygous**	**homozygous**	**homozygous**	**homozygous**	**homozygous**	**compound heterozygous**
	FH-related gen	*LDLR*	*LDLR*	*LDLR*	*LDLR*	*LDLR*	*LDLR*	*LDLR*
	Genome mutation	*c.[500_501delinsTA; 532G* > *A; 728G* > *A]; c.[500_501delinsTA; 532G* > *A; 728G* > *A]*	*c.[1646G* > *A]; c.[1646G* > *A]*	*c.[81C* > *G]; c.[81C* > *G]*	*c.[81C* > *G]; c.[81C* > *G]*	*c.[1773T* > *C]; c.[1773T* > *C]*	*c.[2389G* > *A]; c.[2389G* > *A]*	*c.[1151A* > *C]; c.[1646G* > *A]*
	Exon	*4,4,5*	*11*	*2*	*2*	*12*	*17*	*8,11*
	Protein mutation	*Cys167Leu; Asp178Asn; Cys243Tyr*	*p.Gly549Asp*	*p.Cys27Trp*	*p.Cys27Trp*	*P.Ile585Thr*	*p.Val797Met*	*p.Gly549Asp; p.Gln384Pro*
	Residual LDLR activity	not determined	<2%	15–30%	15–30%	not determined	not determined	p.Gly549Asp < 2% p.Gln384Pro: not determined
Screening type		Other	Other	Universal	Universal	Other	Other	Other
Age of diagnosis (years)		11.1	2.9	6.3	5.5	4.3	12.9	7.3
Symtoms		Xanthomas	Xanthomas	Asymtomatic	Asymtomatic	Xanthomas	Angina pectoris, xanthomas	Xanthomas
Corneal arcus		Yes	No	No	No	No	No	no
Cardiovascular exams		CT coronary angiography and coronary angiogram: normal	Carotid US: diffuse non- obstructive atherosclerosis in both carotid arteries. In right CCA: plaque type I-II (30% stenosis)	No data	Carotid US: cIMT = 0,556 mm	Carotid US: cIMT = 0,903 mm	Coronarography: stenosis of left coronary artery, lower stenosis of right coronary artery	Carotid US: Normal
TC (mmol/l)	First	22.9	15.8	7.6	9.0	24.8	17.2	20.4
	Last	17.5	18.2	7.0	6.0	7.6	16.2	11
	↓	23.4%	−15.2%	7.9%	33.3%	69.4%	5.8%	46.1%
LDL-C (mmol/l)	First	20.5	13.7	No data	7.6	21.6	15.4	18.5
	Last	16.0	17.2	5.5	4.5	6.3	14.4	10.1
	↓	22.0%	−25.5%	–	40.8%	70.8%	6.5%	45.4%
Pharmacotherapy		Atorvastatin 40 mg + Ezetimibe 10 mg	Atorvastatin 80 mg + LDL-apheresis + Ezetimibe 10 mg + *PCSK9* inhibitor	No	Atorvastatin 40 mg + Ezetimibe 10 mg + *PCSK9* inhibitor	Atorvastatin 40mg/simvastatin 30 mg + LDL-apheresis + Liver trasplantation (2004)	Atorvastatin 40 mg + Ezetimibe 10 mg ± LDL-apheresis ± *PCSK9* inhibitor ± Lomitapide	Atorvastatin 80 mg + Ezetimibe 10 mg
Family with hypercholesterolemia		Both parents and brother	Father and grandmother	No	Both parents and brother	Both parents and sister	Both parents and sister	Mother and uncle
Family history of other CVD		Grandfather died of CVD and uncle has had 3 stents. Deceased brother and sister	Grandmother	Not reported	Not reported	Not reported	Father died of MI at 53 years old and mother has CHD	Uncle has CVD with cardiac intervention by-pass at 40 years old

*M, male; F, female; CT, computed tomography; US, ultrasound; CCA, common carotid artery; cITM, carotid intima-media thickness; TC, total cholesterol; LDL-C, low-density lipoprotein-cholesterol; CVD, cardiovascular disease; MI, myocardial infarction; CHD, coronary heart disease.*

## Results

### Demographics

All patients had a family history of early CVD and/or hypercholesterolemia, except patient 3. At the time of the clinical diagnosis, the patient’s median age was 6.3 (2.9–12.9) years. Five patients were symptomatic at the diagnosis (presence of xanthomas and/or corneal arcus), while two were asymptomatic detected through the FH screening program. Four patients were born in Slovenia: both patients born after the implementation of the universal FH screening in this country, were detected through the program; the older two HoFH patients were born prior to the program implementation. The pre-treatment median TC level was 17.2 mmol/L [665 mg/dL] (7.6–24.8 mmol/L) and median LDL-C level was 17.0 mmol/L [655 mg/dL] (7.6–21.6 mmol/L).

In the carotid US, an increased carotid artery intima-media thickness (cIMT) or atherosclerosis signs were found in cases 2, 4 and 5. One patient (case 6) with *angina pectoris* underwent coronarography, detecting stenosis of left and right coronary artery.

### Genotypes

Genetic evaluation confirmed mutations in *LDLR* gene in all of the patients (OMIM #143890). Six patients were confirmed as HoFH and one as cHeFH. The HoFH patients present the mutations p.Gly549Asp, p.Cys27Trp, p.Ile585Thr, p.Val797Met, p.Asp178Asn, p.Cys243Tyr, and p.Cys167Leu (the last three variants are present in each allele of patient 1). Case 7 presents the variants p.Gly549Asp and p.Gln384Pro in each allele (cHeFH) (Table 1). The cascade children-parent testing was performed for patients number 1, 2, 4 and 5, confirming all their parents and some of the siblings are carriers of disease-causing variants (Figure 1). The genetic testing for family members of patients 3, 6 and 7 were unavailable.

### Treatment

As a response to treatment, the median TC and LDL-C decreased by 28 and 31%, respectively. The treatment response is summarized in Figures 2A,B and specified in the Mendeley database^1^. Six patients were treated with statins, five with ezetimibe, three with *PCSK9* inhibitors and one with lomitapide. Three patients received LDL-apheresis. One patient had liver transplantation at the age of 16 with excellent response. It is worth noting the distinguished response presented in the cHeFH patient (46%), compared to the mean response in HoFH patients.

## Discussion

Homozygous FH is an ultra-rare disease, but newer data show it to be more frequent as previously assumed. Sjouke et al. (2014) calculated the prevalence of HoFH and cHeFH in the Netherlands population ranges from 1/371,608 to 1/407,863. The prevalence in Slovenia was estimated at around 1/256,340, calculated by dividing the number of tested children with the number of confirmed HoFH/cHeFH cases in that period.

Up to date, there is no international unanimity on FH detection strategies; some guidelines recommend the cascade screening as the most cost-effective strategy (Santos et al., 2016). Others, like the US National Lipid Association (Goldberg et al., 2011) and American Academy of Pediatrics (Expert Panel on Integrated Guidelines for Cardiovascular Health, and Risk Reduction in Children, and Adolescents, 2011) recommend universal screening. Thus, each country adopts a screening method based on local scientific societies or experts (Ibarretxe et al., 2018; Umans-Eckenhausen et al., 2001; Representatives of the Global Familial Hypercholesterolemia Community et al., 2020). For example, in Sjouke’s report in the Netherlands, 36 patients (73%) were diagnosed by referrals from pediatricians because of the presence of symptoms, while 13 patients (27%) were detected through cascade screening (Sjouke et al., 2014). Slovenia is the only country with implemented nationwide universal FH screening in pre-school children (with routinely implemented genetic FH diagnostics), detecting also the last two of our HoFH patients (the first two were diagnosed prior to the program implementation) (Klancar et al., 2015; Groselj et al., 2018). However, beyond the initial screening method, almost all of the literature agrees on the importance of genetic testing. Recognition of a pathogenic FH mutation guides the cascade screening in the family, as well as the incorporation of genetic testing into cascade screening improves the detection rate for FH (Knowles et al., 2017). Furthermore, between HoFH and cHeFH patients some more subtle differences in the genotype-phenotype correlations or even regarding the response to therapies might exist due to inter-allele interactions and possibility that the phenotype is determined by the allele leading to the higher residual activity (as generally in inborn errors of metabolism) (Groselj et al., 2012).

We reported six patients with HoFH and one with cHeFH. All of them (100%) have mutations in *LDLR*, while other European reports show a prevalence of 91% in Netherlands (Sjouke et al., 2014), 75% in France (Bruckert et al., 2017), and 71% in Spain (Alonso et al., 2016). Our cohort’s mean age at the time of clinical diagnosis was 7.2 years old, ranging from 2.9 to 12.9 years. In other European studies the mean age at the time of molecular diagnosis were: in Austria and France 6.6 and 7.5 years, respectively, in Belgium 2.45 years, while in Netherlands it was 28.2 years (49 cases included, 0–68 years old).

All the patients meet the FH Dutch diagnostic criteria for definite FH, except patient 3 and 4, who had a possible and probable FH, respectively, both detected by universal FH screening (Klancar et al., 2015; Groselj et al., 2018); all patients were genetically diagnosed for FH. Four patients presented xanthomas, whereas in France (Bruckert et al., 2017), Austria (Widhalm et al., 2017), and Belgium (Sanna et al., 2016) almost all of the patients had skin stigmata at the diagnosis.

The pre-treatment mean LDL-C level of our patients was 16.2 mmol/L [627 mg/dL] (but with quite wide range 7.6–21.6 mmol/L). Other European reports mostly showed similar initial LDL-C levels: Netherlands 12.9 mmol/L [498 mg/dL]; Belgium 19.6 mmol/L [757 mg/dL]; Austria 16.5 mmol/L [638 mg/dL] and France 13.2 mmol/L [510 mg/dL] (Sjouke et al., 2014; Sanna et al., 2016; Bruckert et al., 2017; Widhalm et al., 2017).

Regarding CVD, in our cohort four patients showed atherosclerotic signs (plaques in the carotid arteries, increased cITM or coronary stenosis) and one patient (case 6) also presented *angina pectoris*. In the United Kingdom, Thompson et al. (2015) reported that the most frequent complications in these patients were coronary heart disease and aortic stenosis.

Homozygous FH is usually very difficult to manage and the medical treatment often combines several cholesterol-lowering drugs (Cuchel et al., 2014). Initially, statins with ezetimibe are introduced and in responsive patients also *PCSK9* inhibitors, but frequently do not result in a satisfactory reductions of either TC or LDL-C levels, especially in moderate and severe HoFH patients, which have the highest risk of CVD (Kolansky et al., 2008; Rajendran et al., 2013). For over 30 years, LDL-apheresis has been used, becoming a mainstay in the management of the disease and it is currently considered the most safe and effective treatment (Julius, 2017). If LDL-apheresis is not successful, liver transplantation can be an alternative, considering also as a successful therapy in other metabolic liver diseases (McKiernan, 2017). In the Brussels cohort published by Sanna, the median reduction in TC in response to pharmacological treatment was 47% (Sanna et al., 2016). In our cohort, we observed a median decrease of TC levels in response to the combined treatment by 28% (range from a reduction of 69% to an increase of 15% besides the therapy). A huge variability in the treatment response is observed among our patients (Figures 2A,B). We could recognize the differences depending on the approach to therapies and on patient adherence, such as cases 2 and 6. In developing countries, the access to modern diagnostic and therapeutic methods is still limited, resulting in patients failure in reaching treatment goals and also in inadequate management of CVD (Representatives of the Global Familial Hypercholesterolemia Community et al., 2020).

**FIGURE 3:**
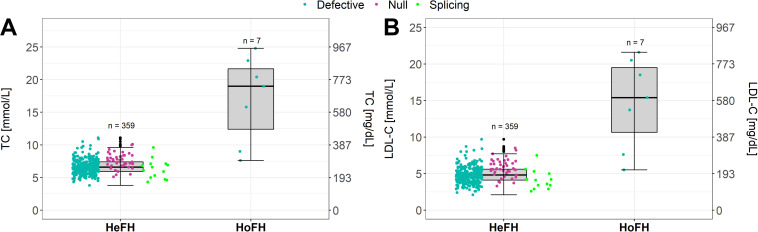
**(A,B)** Variability in HoFH (real HoFH and cHeFH) from HeFH patients in CT and LDL-C levels.

Groselj et al. (2018) reported that around 45% of participants referred from the Slovenian universal FH screening presented a disease-causing genetic variant for FH, mostly heterozygous. Our institution could compare the TC and LDL-C levels of HeFH patients with the HoFH/cHeFH patients (Figures 3A,B). In concordance with the literature (Santos et al., 2016; Sturm et al., 2018; Berberich and Hegele, 2019), our cohort shows to be clinically and genetically very diverse, overlapping with HeFH phenotypes. The clinical criteria (as Simon Broome criteria) might be applicable already in children to raise suspicion of FH but in some cases fail to distinguish HeFH and HoFH/cHeFH patients. As reported previously, consideration of a diagnosis of HoFH/cHeFH should not be limited to those with very high LDL-C levels (Raal et al., 2016). Other factors besides Mendelian inheritance also play a role in the FH: polygenic variants, gene-environment interactions and non-mendelian mechanisms, such as epigenetic (Hooper et al., 2018; Berberich and Hegele, 2019).

Because of the retrospective design, our report has some limitations in data interpretation and not all the data of individual patients or their relatives were available to be included.

## Conclusion

HoFH/cHeFH patients are clinically heterogeneous, possibly even overlapping with HeFH patients, highlighting the importance of establishing the genetic diagnosis. In addition, public policies are needed to improve early detection, family screening, adequate therapies, and appropriate follow-up. HoFH/cHeFH patients in developing countries frequently lack even basic access to diagnostics, management and adequate therapeutic options, leading to the inequality of outcomes. This should be better addressed at the global level.

## Patients Perspective

Nowadays, in Slovenia patients have good access to genetic diagnosis, early detection (universal FH screening) and the current methods of treatment, which is not the case in Kosovo and in Pakistan.

## Data Availability Statement

The datasets presented in this study can be found in online repositories. The names of the repository/repositories and accession number(s) can be found in the manuscript/supplementary material.

## Ethics Statement

Written consent was obtained from the participants to conduct clinical examination, conducting clinical and laboratory investigations, including genetic studies and publishing the patient’s data in scientific journals.

## Author Contributions

UG and TM conceptualized and designed the study, performed the clinical work, carried out the initial analyses, drafted the initial manuscript, and reviewed and revised the manuscript. US, MM, MC, JK, FS, SS, IK, and VK performed the clinical work, designed the data collection instruments, and reviewed and revised the manuscript. TB and KT coordinated and supervised data collection, and critically reviewed the manuscript for important intellectual content. All authors approved the final manuscript as submitted and agree to be accountable for all aspects of the work.

## Conflict of Interest

The authors declare that the research was conducted in the absence of any commercial or financial relationships that could be construed as a potential conflict of interest.

## Footnotes

1 http://dx.doi.org/10.17632/thpt9htws6.1

## References

[B1] Ahmed W. Whittall R. Riaz M. Ajmal M. Sadeque A. Ayub H. (2013). The genetic spectrum of familial hypercholesterolemia in Pakistan. *Clin. Chim. Acta* 421 219–225. 10.1016/j.cca.2013.03.017 23535506 PMC3701840

[B2] Ajmal M. Ahmed W. Sadeque A. Benish Ali S. H. Bokhari S. H. Ahmed N. (2010). Identification of a recurrent insertion mutation in the LDLR gene in a Pakistani family with autosomal dominant hypercholesterolemia. *Mol. Biol. Rep.* 37 3869–3875. 10.1007/s11033-010-0043-0 20217239

[B3] Alonso R. Diaz-Diaz J. L. Arrieta F. Fuentes-Jiménez F. De Andres R. Saenz P. (2016). Clinical and molecular characteristics of homozygous familial hypercholesterolemia patients: insights from SAFEHEART registry. *J. Clin. Lipidol.* 10 953–961. 10.1016/j.jacl.2016.04.006 27578128

[B4] Benito-Vicente A. Uribe K. B. Jebari S. Galicia-Garcia U. Ostolaza H. Martin C. (2018). Validation of LDLr activity as a tool to improve genetic diagnosis of familial hypercholesterolemia: a retrospective on functional characterization of LDLr variants. *Int. J. Mol. Sci* 19:1676. 10.3390/ijms19061676 29874871 PMC6032215

[B5] Berberich A. J. Hegele R. A. (2019). The complex molecular genetics of familial hypercholesterolaemia. *Nat. Rev. Cardiol.* 16 9–20. 10.1038/s41569-018-0052-6 29973710

[B6] Bruckert E. Kalmykova O. Bittar R. Carreau V. Béliard S. Saheb S. (2017). Long-term outcome in 53 patients with homozygous familial hypercholesterolaemia in a single centre in France. *Atherosclerosis* 257 130–137. 10.1016/j.atherosclerosis.2017.01.015 28131047

[B7] Cuchel M. Bruckert E. Ginsberg H. N. Raal F. J. Santos R. D. Hegele R. A. (2014). Homozygous familial hypercholesterolaemia: new insights and guidance for clinicians to improve detection and clinical management. A position paper from the consensus panel on Familial Hypercholesterolaemia of the European Atherosclerosis Society. *Eur. Heart J.* 35 2146–2157. 10.1093/eurheartj/ehu274 25053660 PMC4139706

[B8] Diakou M. Miltiadous G. Xenophontos S. Cariolou M. Heta N. (2010). Characterization of low density lipoprotein receptor (LDLR) gene mutations in Albania. *Arch. Med. Sci.* 6 198–200. 10.5114/aoms.2010.13894 22371747 PMC3281340

[B9] EAS Familial Hypercholesterolaemia Studies Collaboration, Vallejo-Vaz A. J. De Marco M. Stevens C. A. Akram A. Freiberger T. (2018). Overview of the current status of familial hypercholesterolaemia care in over 60 countries - The EAS Familial Hypercholesterolaemia Studies Collaboration (FHSC). *Atherosclerosis* 277 234–255. 30270054 10.1016/j.atherosclerosis.2018.08.051

[B10] Expert Panel on Integrated Guidelines for Cardiovascular Health, and Risk Reduction in Children, and Adolescents (2011). Expert panel on integrated guidelines for cardiovascular health and risk reduction in children and adolescents. Summary report. *Pediatrics* 128 213–256. 10.1542/peds.2009-2107C PMC4536582 22084329

[B11] Goldberg A. C. Hopkins P. N. Toth P. P. Ballantyne C. M. Rader D. J. Robinson J. G. (2011). Familial hypercholesterolemia: screening, diagnosis and management of pediatric and adult patients: clinical guidance from the national lipid association expert panel on familial hypercholesterolemia. *J. Clin. Lipidol.* 5 1–8. 10.1016/j.jacl.2011.04.003 21600517

[B12] Groselj U. Kovac J. Sustar U. Mlinaric M. Fras Z. Trebusak Podkrajsek K. (2018). Universal screening for familial hypercholesterolemia in children: the Slovenian model and literature review. *Atherosclerosis* 277 383–391. 10.1016/j.atherosclerosis.2018.06.858 30270075

[B13] Groselj U. Zerjav Tansek M. Kovac J. Hovnik T. Trebusak Podkrajsek K. Battelino T. (2012). Five novel mutations and two large deletions in a population analysis of the phenylalanine hydroxylase gene. *Mol. Genet. Metab.* 106 142–148. 10.1016/j.ymgme.2012.03.015 22513348

[B14] Hooper A. J. Burnett J. R. Bell D. A. Watts G. F. (2018). The present and the future of genetic testing in familial. hypercholesterolemia: opportunities and caveats. *Curr. Atherosclerosis Rep.* 20:31. 10.1007/s11883-018-0731-0 29779130

[B15] Ibarretxe D. Rodríguez-Borjabad C. Feliu A. Bilbao J. A. Masana L. Plana N. (2018). Detecting familial hypercholesterolemia earlier in life by actively searching for affected children: the DECOPIN project. *Atherosclerosis* 278 210–216. 10.1016/j.atherosclerosis.2018.09.039 30312929

[B16] Julius U. (2017). History of lipidology and lipoprotein apheresis. *Atheroscler. Suppl.* 30 1–8. 10.1016/j.atherosclerosissup.2017.05.034 29096824

[B17] Khan S. P. Ghani R. Yaqub Z. (2014). Single step PCR for the identification of Low Density Lipoprotein Receptor (LDL-R) gene mutations. *Pak. J. Med. Sci.* 30 830–833. 10.12669/pjms.304.4711 25097526 PMC4121707

[B18] Klancar G. Groselj U. Kovac J. Bratanic N. Bratina N. Trebusak Podkrajsek K. (2015). Universal screening for familial hypercholesterolemia in children. *JACC* 66 1250–1257. 26361156 10.1016/j.jacc.2015.07.017

[B19] Knowles J. W. Rader D. J. Khoury M. J. (2017). Cascade screening for familial hypercholesterolemia and the use of genetic testing. *JAMA* 318 381–382. 10.1001/jama.2017.8543 28742895 PMC6166431

[B20] Kolansky D. M. Cuchel M. Clark B. J. Paridon S. McCrindle B. W. Wiegers S. E. (2008). Longitudinal evaluation and assessment of cardiovascular disease in patients with homozygous familial hypercholesterolemia. *Am. J. Cardiol.* 102 1438–1443. 10.1016/j.amjcard.2008.07.035 19026292

[B21] Masana L. Ibarretxe D. Rodríguez-Borjabad C. Plana N. Valdivielso P. Pedro-Botet J. (2019). Toward a new clinical classification of patients with familial hypercholesterolemia: one perspective from Spain. *Atherosclerosis* 287 89–92. 10.1016/j.atherosclerosis.2019.06.905 31238171

[B22] McKiernan P. J. (2017). Recent advances in liver transplantation for metabolic disease. *J. Inherit. Metab. Dis.* 40 491–495. 10.1007/s10545-017-0020-z 28168361

[B23] Moorjani S. Roy M. Torres A. Bétard C. Gagné C. Lambert M. (1993). Mutations of low-density-lipoprotein-receptor gene, variation in plasma cholesterol, and expression of coronary heart disease in homozygous familial hypercholesterolaemia. *Lancet* 341 1303–1306. 10.1016/0140-6736(93)90815-x 8098448

[B24] Raal F. J. Sjouke B. Hovingh K. Isaac B. J. (2016). Phenotype diversity among patients with homozygous familial hypercholesterolemia: a cohort study. *Atherosclerosis* 248 238–244. 10.1016/j.atherosclerosis.2016.03.009 27017151

[B25] Rajendran R. Srinivasa K. H. Rangan K. Hegde M. Ahmed N. (2013). Supravalvular aortic stenosis in a patient with homozygous familial hypercholesterolaemia. *Eur. Heart J. Cardiovasc. Imaging* 14:1023. 10.1093/ehjci/jet072 23793874

[B26] Representatives of the Global Familial Hypercholesterolemia Community, Wilemon K. A. Patel J. Aguilar-Salinas C. Ahmed C. D. Alkhnifsawi M. (2020). Reducing the clinical and public health burden of familial hypercholesterolemia: a global call to action. *JAMA Cardiol.* 5 217–229. 31895433 10.1001/jamacardio.2019.5173

[B27] Sanna C. Stephenne X. Revencu N. Smets F. Sassolas A. Di Filippo M. (2016). Homozygous familial hypercholesterolemia in childhood: genotype- phenotype description, established therapies and perspectives. *Atherosclerosis* 247 97–104. 10.1016/j.atherosclerosis.2016.02.009 26894473

[B28] Santos R. D. Gidding S. S. Hegele R. A. Cuchel M. A. Barter P. J. Watts G. F. (2016). Defining severe familial hypercholesterolaemia and the implications for clinical management: a consensus statement from the international atherosclerosis society severe familial hypercholesterolemia panel. *Lancet Diab. Endocrinol.* 4 850–861. 10.1016/S2213-8587(16)30041-9 27246162

[B29] Sjouke B. Hovingh G. K. Kastelein J. J. Stefanutti C. (2015). Homozygous autosomal dominant hypercholesterolaemia: prevalence, diagnosis, and current and future treatment perspectives. *Curr. Opin. Lipidol.* 26 200–209. 10.1097/mol.0000000000000179 25950706

[B30] Sjouke B. Kusters D. M. Kindt I. Besseling J. Defesche J. C. Sijbrands E. J. (2014). Homozygous autosomal dominant hypercholesterolaemia in The Netherlands: prevalence, genotype-phenotype relationship, and clinical outcome. *Eur. Heart J.* 36 560–565. 10.1093/eurheartj/ehu058 24585268

[B31] Sturm A. C. Knowles J. W. Gidding S. S. Ahmad Z. S. Ahmed C. D. Ballantyne C. M. (2018). Clinical genetic testing for familial hypercholesterolemia JACC scientific expert panel. *JACC* 72 662–680. 30071997 10.1016/j.jacc.2018.05.044

[B32] Thompson G. Seed M. Naoumova R. P. Neuwirth C. Walji S. Aitman T. J. (2015). Improved cardiovascular outcomes following temporal advances in lipid-lowering therapy in a genetically-characterised cohort of familial hypercholesterolaemia homozygotes. *Atherosclerosis* 243 328–333. 10.1016/j.atherosclerosis.2015.09.029 26433113

[B33] Umans-Eckenhausen M. A. Defesche J. C. Sijbrands E. J. Scheerder R. L. Kastelein J. J. (2001). Review of first 5 years of screening for familial hypercholesterolaemia in the Netherlands. *Lancet* 357 165–168. 10.1016/s0140-6736(00)03587-x 11213091

[B34] Vallejo-Vaz A. J. Kondapally Seshasai S. R. Cole D. Hovingh G. K. Kastelein J. J. Mata P. (2015). Familial hypercholesterolaemia: a global call to arms. *Atherosclerosis* 243 257–259. 26408930 10.1016/j.atherosclerosis.2015.09.021

[B35] Watts G. F. Gidding S. Wierzbicki A. S. Toth P. P. Alonso R. Virgil Brown W. (2014). Integrated guidance on the care of familial hypercholesterolaemia from the international FH foundation: executive summary. *J. Atheroscler. Thromb.* 21 368–374. 24892180

[B36] Widhalm K. Benke I. M. Fritz M. Geiger H. Helk O. Fritsch M. (2017). Homozygous familial hypercholesterolemia: summarized case reports. *Atherosclerosis* 257 86–89. 10.1016/j.atherosclerosis.2017.01.002 28126585

